# Diagnosis of cancer, autoimmune and infectious diseases and prediction of the therapy effectiveness based on the individual’s immunotype

**DOI:** 10.3389/fimmu.2025.1658970

**Published:** 2025-08-29

**Authors:** Dmitry V. Tabakov, Anna A. Maznina, Ekaterina A. Astakhova, Anastasia E. Egorova, Elena N. Zakharova, Olga V. Glushkova, Ekaterina S. Petriaikina, Dmitry V. Svetlichnyy, Julia A. Krupinova, Viktor P. Bogdanov, Vladimir S. Yudin, Anton A. Keskinov, Sergey M. Yudin, Mary Woroncow, Veronika I. Skvortsova, Pavel Yu Volchkov

**Affiliations:** ^1^ Federal State Budgetary Scientific Institution “Federal Research Center for Innovator and Emerging Biomedical and Pharmaceutical Technologies”, Moscow, Russia; ^2^ Moscow Center for Advanced Studies, Moscow, Russia; ^3^ Federal State Budgetary Scientific Institution “Petrovsky National Research Centre of Surgery”, Moscow, Russia; ^4^ Federal State Budgetary Institution «Centre for Strategic Planning and Management of Biomedical Health Risks» of the Federal Medical and Biological Agency (Centre for Strategic Planning, of the Federal Medical and Biological Agency), Moscow, Russia; ^5^ Moscow Clinical Scientific Center named after A.S.Loginov, Moscow, Russia; ^6^ Lomonosov Moscow State University, Moscow, Russia; ^7^ The Federal Medical Biological Agency (FMBA of Russia), Moscow, Russia

**Keywords:** immune system, immunotype, autoimmune disease, cancer, immunophenotyping

## Abstract

Immune system plays a central role in the pathogenesis of cancer and autoimmune diseases. An entire field has emerged to identify separate minor cell subpopulations carrying potential molecular targets or activation markers to study their prognostic role in disease progression and severity or predictive potential to use immunotherapy. However, the biomarker potential of minor populations is limited, as it does not take into account systemic interactions between populations of the immune system. A number of studies in the COVID era have shown that the certain balance between immune cell populations in donor’s blood, called ‘immunotype’, can predict the outcome of treatment and the onset of a cytokine storm. This observation was extended to other diseases, including cancer and autoimmunity. It was shown that the immunotype can be used to diagnose both the presence of the disease itself, as well as its form or progression, to stratify patients in the risk groups and to predict the effectiveness of therapy. The most important advantages of immunotype-based diagnostics are its low invasiveness, the possibility of multiple biomaterial sampling, and the complexity of the analysis by the simultaneous assessment of blood cell composition and their functional activity. In this review, we summarize currently available studies of immunotypes and defined key subpopulations, their possible impact in diagnostics and personalization of the therapy in clinical routine practice in various diseases.

## 1 Introduction

At present, there is a consensus on the central role of the immune system in the pathogenesis of cancer and autoimmune diseases (1, 2). This idea has served as a basis for more profound study of the structure of immunity and the identification of individual populations or markers of immunocompetent cells that directly influence the development of the disease. The most obvious example is the checkpoint molecules - markers capable of activating or inhibiting the immune response when stimulated by a specific ligand. For instance, presence of PD-1 expression on the surface of lymphocytes is a target for anti-PD-1 therapy with nivolumab or pembrolizumab in different forms of cancer (3, 4). CTLA-4, CD40, ICOS, Lag-3, GITR, OX40, CD28, and many other cell surface markers are also сheckpoint molecules, for which stimulation effects have already been described and drugs based on monoclonal antibodies that block or, on the contrary, activate their downstream signals have been created (5, 6).

It should be noted that, despite autoimmune diseases and cancer being opposite from an immunological point of view (in the first case there is an overactivation of the immune system, in the second case - it’s systemic or local suppression), the concept of immune checkpoints has enabled us to develop tools to influence immunomodulation using the same molecular targets (7). Thus, drugs to the same checkpoints that have opposite methods of action have been described (e.g., nivolumab, which blocks the suppressor effect of the PD-1 molecule developed for cancer therapy (8), and CC-90006, a PD-1 agonist that induces suppression of the immune response for psoriasis therapy (9). The same is true for other molecules in the checkpoint category (8, 10).

The quantitative assessment of the expression of a specific molecule on tumor cells or on the surface of an immunocompetent cell, assessed by immunohistochemistry or flow cytometry, is a predictor for the use of immunotherapy (11, 12). In this regard, an entire field has emerged to identify cell subpopulations carrying potential molecular targeting or activation markers to study their prognostic role in assessing disease progression and severity. A huge number of minor subpopulations with statistically significant correlation both with disease progression, its dynamics, and prediction of therapy efficacy (especially immunotherapy) have been identified (13–15).

While it seems logical to assess the expression of a specific marker when administering a targeted drug, the relationship of one specific population to the course of disease is highly contentious. The immune system is a highly heterogeneous and dynamically changing system where multiple populations have plasticity and can change their functional activity depending on a combination of factors presented at the systemic and local levels (16). The interconversion of M1/M2 populations of macrophages (17) and MDSCs (18), the repurposing of the Treg population into IL-17 producers in T2D (19), and the possibility of activation of effector populations with an exhausted phenotype (20) may serve as vivid examples. Thus, it is rather difficult to assess the role of a specific population without understanding the context of the immune response in a given case. For example, patients with high immune infiltration in tumor tissue are known to have better survival in cancer (21). However, if most of them have an exhausted phenotype, successful therapy requires not only triggering the immune response by blockade of a single suppressor mechanism, but also providing an influx of naive T lymphocytes capable of developing a cytotoxic response (22, 23). In this case, peripheral blood acts as a source of immune system reserves, and therefore it would be reasonable to assess the potential and consistency of systemic immunity - to evaluate the pool of naive and effector cells, to assess the ratio of effector and suppressor components (24). The same approach is applicable to autoimmune diseases - in T1D a decrease in Treg penetration into pancreatic tissues has been described. If there is a defect in the development of this population itself or its functional failure (e.g., decreased expression of CD39/CD73 system, CTLA4, FoxP3), targeted immunotherapy aimed at attracting Treg cells or activating its suppressor capacity will fail (25).

In order to assess the full context of the processes occurring in the immune system at a given time point, the concept of immunotypes - clustering of patients (suffering from the same disease) depending on the ratio of key subpopulations of the immune system - is becoming more widely used. Flow cytometry, bulk RNAseq, and scRNAseq of peripheral blood cells, lymph nodes, tissues, tumors, and foci with autoimmune lesions are widely used for a comprehensive assessment of the immune system (26, 27).

**Figure 1:**
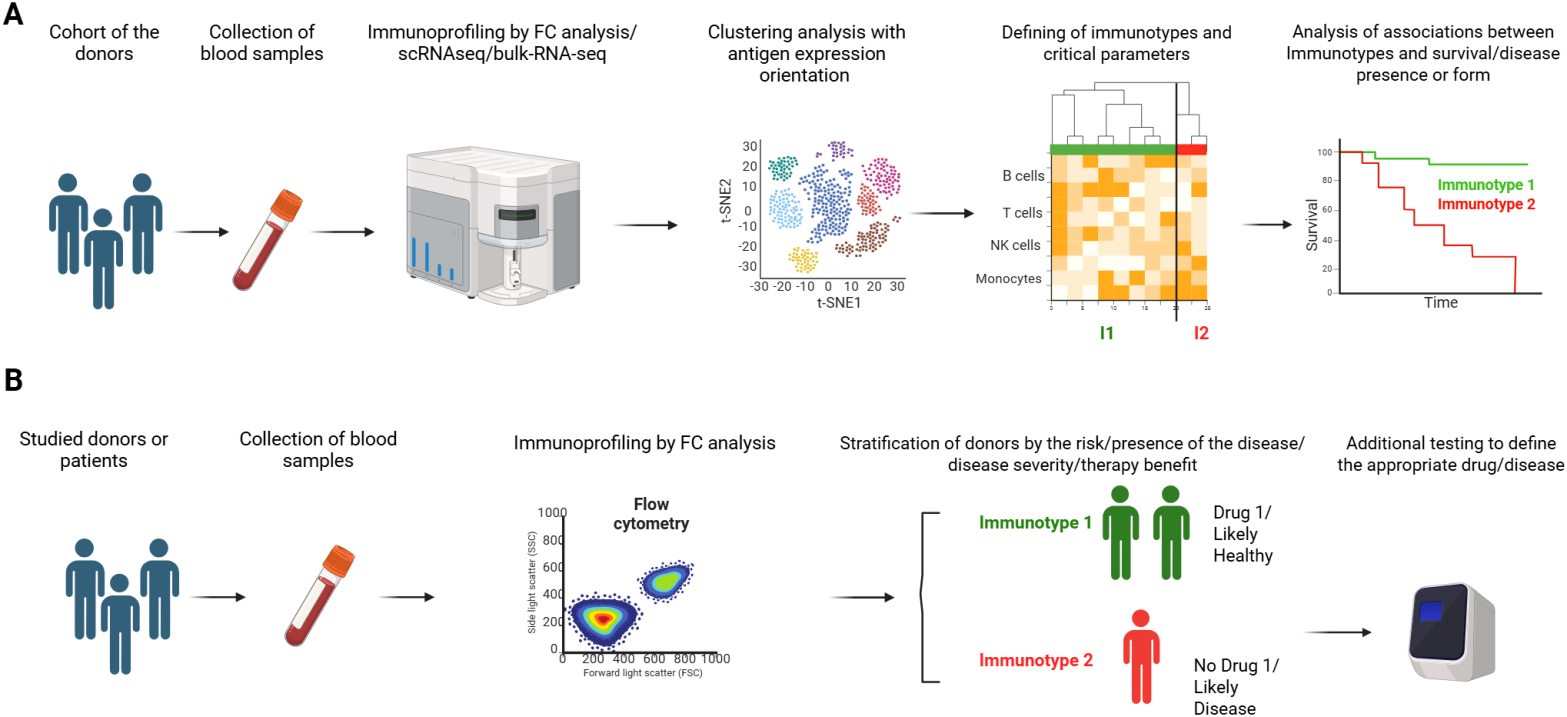
Possible application of immunotype concept. **(A)** Research step. Defining of immunotypes and their optimal number present in the cohort with the same condition, identification of critical parameters for immunotype stratification, assessment on survival or prognostic impact. **(B)** Clinical use of defined immunotypes by patient/donor stratification.

The huge advantages of immunotyping of peripheral blood are the high availability of biomaterial, the possibility of regular monitoring and the completeness of the study. These properties make immunotyping a promising tool in healthcare, as they make it possible, based on a blood test, to divide donors by the risk of developing the disease, indicate the possibility of its presence or its specific form, and predict the effectiveness of therapy. Due to the simplicity of its use (since it is possible to donate blood for such an analysis at any time during a routine checkup), immunotyping can become one of the starting points for differential diagnosis in routine clinical practice (**Figure 1**). In another hand, immunotyping allows the assessment of the contribution of a particular state of the immune system as a whole to the development of a disease or immune responses to a particular pathological process, and to identify new predictive and prognostic biomarkers.

Here, we provide examples of donor stratification based on immune status and how determining this status on peripheral blood may impact therapeutic decisions or predicting disease outcome.

## 2 Immunotypes of healthy individuals

At the start, the purpose of identifying immunotypes of healthy donors was to assess the influence of age, gender, infection, and vaccination status on the subpopulation structure of the immune system. Studying the immunotypes of healthy donors helps establish the ‘reaction norm’ for the ratios of different immune populations, enabling future comparisons with data obtained from patients suffering from disease.

In 2017, Kaczorowski et al. (28) analyzed 1546 samples from healthy donors and presented a possible approach to clustering samples and identifying immunotypes. This study was the first attempt to apply a donor clustering approach to define any dependencies between immune status and different variables such as age, gender, and genetic factors. One of the primary findings is that the Euclidean distance between points representing twins was smaller than that between unrelated donors, indicating a partial association of the subpopulational structure of the immune system with genetic factors. The authors defined key combinations of immune populations to predict response to immune system stimulation. The differences between these feature combinations were highly associated with, but not predetermined by, age, gender, and CMV seropositivity. Moreover, logical assumptions about the role of CD4/CD8 ratios or the centrality of lineage populations in immunotype formation (monocytes, T lymphocytes, NK cells) did not hold; samples with marked imbalances in such populations were not clustered separately. Thus, it has been hypothesized that individual immune cell measurements alone do not identify individuals with unusual phenotypes, and only by analyzing their overall balance of immune cell subsets can such donors be identified. Researchers have shown that the diversity of possible immunotypes increases in the aging subset compared with younger donors if clustering was performed separately, and this suggests the immune system is more homogeneous in healthy donors at a young age. The same observation was noted in monozygotic twins (29).

Unfortunately, the authors did not specify which parameters in the balance of immune populations were stratifying the immunotypes, limiting themselves to listing all the analyzed populations. Nevertheless, this study showed a possible effect of using clustering of donors by immunotypes and identifying associations with various parameters or directions of immune response development. This led to the further development of this area and the extrapolation of the approach to various diseases and a deeper study of the immune landscape of healthy donors.

Cevirgel et al. (30) found 9 immunotypes in 318 healthy donors. The difference between immunotypes was largely determined by T lymphocyte subpopulations, including the prevalence of naive or memory subpopulations. Only one immunotype (#8) was unambiguously associated with an increased presence of B lymphocytes, two more (2 and 3) were partially associated with an increased content of classical monocytes (**Table 1**).

**Table 1:** - Immunotypes described in Cevirgel et al. study.

Immunotype	CMV+ (%)	Immune subsets (high)	Immune subsets (low)	Association
1	49%	CD8+ True naive	CD4+ Treg, CD4+ CD95+, CD8+ CD95+, CD4+ Tcm, CD8+ Tcm	Younger age
2	77%	Classical monocytes, CD4+ HLA-DR+, CD4+ Tcm, CD8+ Tem	CD8+ True naive	Early signs of immune system aging, CMV positivity
3	10%	CD8+ True naive, classical monocytes, CD4+ Tscm	CD4+ CD95+	Younger age
4	19%	CD8+ True naive, CD4+ HLA-DR+, CD4+ Tcm	CD4+ Treg	No clear associations
5	90%	CD4+ Tscm	CD8+ True naive, classical monocytes, CD4+ HLA-DR+, CD4+ Tcm, CD8+ Tcm	aging‐associated phenotype, CMV positivity
6	16%	CD8+ True naive, CD4+ Tscm, CD8+ Tscm	CD4+ HLA-DR+, CD4+ Tcm	aging‐associated phenotype
7	61%	CD4+ Treg, CD4+ CD95+, CD8+ CD95+, CD4+ HLA-DR+, CD4+ Tcm, CD8+ Tem	CD8+ True naive	Remodeling
8	88%	CD19+ CD95+	CD8+ True naive, CD4+ Tscm, CD8+ Tscm	Remodeling, CMV positivity
9	19%	CD8+ CD95+, CD8+ Tscm, CD4+ Tcm, CD19+ CD95+	CD8+ True naive	Remodeling

This study was able to establish an association between a specific immunotype and age. Immunotypes 1 and 3, which pertain to younger individuals, showed the the highest percentage of CD4+ true naive and CD8+ true naive T cells.

The authors hypothesized that immunotype 6, with a similar percentage of true naive CD4+ T cells, but comprised of older individuals, represented a cluster of immunologically healthy donors. Meanwhile, immunotypes 7, 8, and 9 (mean age > 70 years) may be the result of a high degree of immune system remodeling.

Interestingly, immunotype 2, despite being the second youngest immunotype in terms of mean age, showed higher levels of HLA-DR+CD4+ cells than immunotypes 1, 3, 5, and 6, and fewer true naive CD4+ cells, which may indicate early signs of a phenotype associated with immune system aging. In addition, the authors examined the effect of immunotype on immune system stability during vaccination. Immunotypes 1, 5 and 6 were the most stable one year after vaccination, while immunotypes 2, 7, 8 and 9 had the lowest stability.

The authors attributed these differences to the number of HLA-DR+CD4+ and HLA-DR+ CD8+ T cells, which differed between these clusters. When analyzing the response to influenza vaccination, immunotypes 1, 5, and 6, which were characterized by high stability, showed a strong increase in CD4+CD38+ Tfh cells on day 7 after vaccination, in contrast to immunotypes with low stability. These observations suggest that the stability of the immune system over time may be a feature that is related to the composition of immune cell subsets and immune activation of cell subsets associated with vaccination.

Subsequently, as a continuation of the previous study, Cevirgel et al. developed the idea by comparing immunotype identity to the responses to Quadrivalent Inactivated Influenza Vaccine (QIV), Prevenar 13 (PCV13) and SARS-CoV-2 vaccines (mRNA-1273 or BNT162b2) vaccines (31). Immunotype 1 was associated with increased odds of belonging to a higher quartile of triple vaccine response. In addition, individuals with immunotype 6 also had significantly increased odds of having a higher quartile of triple vaccine sensitivity. Conversely, individuals assigned to immunotype 8 had significantly lower sensitivity to the triple vaccine. In a separate ordinal logistic regression model, CMV seropositivity per se was not associated with response to the triple vaccine.

The role of CMV in immunotype formation is a matter of contention, as well. Some researchers have emphasized age as the primary factor during which the number of CMV-seropositive donors increases, and have linked the formation of a particular immunotype to aging. Kaczorowski et al. divided the cohort into several age groups and compared CMV-seropositive and seronegative donors within them. CMV-seropositive donors were found to have a profile closer to the “aging” immunotype even in groups with younger participants (28).

In the study by Cevirgel et al. two immunotypes (5 and 8) contained 91 and 88% CMV-seropositive donors. These immunotypes are characterized by increased CD4+ Tscm and CD95+ B lymphocytes, respectively, with decreased naive T cell subpopulations. These ratios reflect the activation of the immune system and logically explain the results of such clustering. In contrast, clusters with low CMV content (3, 4 and 6) were characterized by prevalence of naive T lymphocytes (31).

## 3 Immunotypes of autoimmune diseases

### 3.1 Type 1 diabetes mellitus

Type 1 diabetes is an autoimmune disease, when insulin-producing beta cells are destroyed by the immune system. Analyses of pancreas sections harvested from individuals with T1D have shown fulminant immune infiltration within individual islets, corroborating a key role for CD4+ and CD8+ T cells (32), as well as NK (33) in beta cell destruction.

Shapiro et al. (34) applied clustering of patients by immune system indicators of peripheral blood in type 1 diabetes mellitus. Initially, the authors demonstrated that the trends in immune profiles in T1D patients were generally similar to healthy donors (HD) and divided into 4 clusters. However, direct comparison revealed an average upward shift in cluster 1 and a downward shift in cluster 3 for T1D trajectories, and this fact allows to suggest that T1D patients demonstrate accelerated aging changes. The trajectories tended to shift further apart over time, which collectively suggests that there are distinguishable, somewhat distinct age-related changes in immune trajectories in people with T1D. The authors found that T1D patients showed some acceleration of immune system aging, unrelated to CMV seropositivity, glycated hemoglobin levels, and genetic risk of developing T1D (as measured by the GRS1 scale). Although the researchers did not identify individual clusters of immune composition in T1D patients, they did develop a model for predicting T1D while correcting for the age-related changes identified. This enabled a clear separation of the cluster of T1D patients from HD and relatives of T1D patients based on the analysis of 48 parameters, with a prediction accuracy of 82.3%. The greatest mean difference between T1D and healthy donors (without significant age dependence) was a significant increase in the frequency of CXCR3 expression among naive CD8+ T cells/altered expression of the co-inhibitory PD-1 receptor in subgroups of T cells. Despite a significantly increased frequency of PD-1+ cells in naive CD4+, naive CD8+, and CD8+ Temra subgroups in T1D participants, PD-1 expression intensity (MFI) was reduced in T1D participants in most subgroups analyzed: CD4+ Tem, CD4+ Temra, CD4+ Tcm and CD8+ Tcm. Interestingly, MFI PD-1 was also significantly reduced in the CD4+ and CD8+ memory T cell subsets of negative autoantibodies (AAb-) relatives compared to HD, suggesting a potential genetic predisposition to altered PD-1 expression. HLA-DR MFI was elevated in participants heterozygous for HLA-DR4 compared to those carrying other HLA class II genotypes (DRX/X), and further increased in participants homozygous for HLA-DR4. Notably, the association between HLA-DR4 genotype and HLA-DR MFI on monocytes was present in all groups, suggesting that this genetic factor of immune phenotype may act independently of AAb positivity or disease status.

Larsson et al. also applied clustering to identify differences between the immunotypes of healthy donors and T1D patients. When comparing clustering between T1D patients and healthy donors, differences in the size and intensity of CD4+ and CD8+ T cell, B cell, NK cell, monocyte, and eosinophil clusters were observed (35). Researchers have found that an increase of activated Tregs, activated CD4+ T cells, activated CD8+ T cells, CD4+CD8+ T cells, Th1 T cells, Th17 T cells and central memory CD8+ T cells was associated with the presence of T1D. In addition, there were several clusters associated with healthy subjects, namely Th2 T cells and naive CD4+ T cells. Cluster analysis revealed lower levels of galectin-10+ eosinophils and higher levels of immature eosinophils in patients with type 1 diabetes mellitus.

A 2024 study by Honardoost et al. (35) found that patients with type 1 diabetes had a number of immunophenotype differences from healthy controls, namely increased Mo/cDC cells, naive and effector T cells, CD8+ naive T cells, and C-monocytes and pDC; decreased lymphoid cells, CD4+ effector memory (EM) cells, Treg cells. Additionally, the ratio of CD4+ and CD8+ cells to Treg cells was increased in T1D patients. Notably, this study, which was conducted on 46 stage 3 T1D patients, and 31 matched controls found that presence of HLA risk types had absolutely no effect on clusterization in both patients and controls.

This study focused on assigning risk factors (TMZ score) to cell types through their differentially expressed genes (DEGs), allowing patients to be clustered into groups based on the severity of the DEG of their PBMCs. TMZ score was T1DM metagene z-score, which was assigned in such a way as to allow for the differentiation between patient subtypes, as well as between patients and controls. 29/31 controls were in the low-response group (low activity of immune response), as were 14/46 T1D patients, suggesting they may have a milder systemic immune response. The intermediate and high response groups were 33/35 T1D cases, with the high response group including a single control patient, who was diagnosed with T1D 4 years after blood was collected for the research.

Verapamil, abatacept, and rituximab, three of the drugs used on patients within this cohort after initial blood sampling, were found to reduce TMZ score, while Teplizumab did not. Additionally, DEGs in adaptive immune cells were found to be significantly enriched in patients with T1D genetic risks. B cell DEGs showed the highest correlation with heritability, largely driven by HLA locus risks, which is notable, as HLA locus risks in themselves did not affect clusterization.

A different 2024 study by Starskaia et al. (36) explored the effects of first-appearing autoantibodies in children with T1D on their overall immunotype. They found that children that had 2 or more autoantibodies initially appear had increased NK cells and γδ+ T cells, and decreased CD4+ and CD8+ T cells, the latter of which was associated with disease progression. In patients where GADA autoantibodies were first present, the populations of NK and CD8+ T cells were increased and the B cell subset was decreased. There were also significant differences in NKT, MAIT cells, and mDCs observed, however the low abundance of these cells in children means further research is required for any true conclusion to be reached about their populations. Children that initially presented with IAA autoantibodies had elevated CD39 levels on CD4+ cells than in the controls. Such an increase was observed on the CD25+CD127– subpopulation with the memory Treg phenotype and in HLA-DR+ICOS+ T cells.

The other important finding of this study is identifying CD161 elevated levels on NK cells in 2 and more AAb groups compared to control. The difference was prominent at the very early stage of disease development, before seroconversion. At the same time, the expression levels of CD27, a marker associated with immature phenotype of NK cells, were lower cases of ≥2 AAb first group than in controls. Another important feature of the group with multiple AAbs was a decrease of inhibitory molecule TIGIT expression on the subsets of CD4+ cells. Based on these findings, we can conclude that the appearance of the multiple Aab phenotype is associated with active proinflammatory processes which are revealed as increase of effector populations and decrease of regulatory subsets.

This study is important because it shows the applicability of the division into immunotypes for predicting the phenotype of diabetes mellitus and the time of its manifestation. Potentially, the concept of immunotypes would be useful for determining changes in the immune status of T cells before seroconversion and can improve early diagnostics of T1D during monitoring study of genetically predisposed individuals.

### 3.2 Kawasaki disease

Kawasaki disease (KD) is considered a kind of systemic vasculitis syndrome, and it primarily invades the medium-sized muscular arteries. Histologically, coronary arteritis begins 6–8 days after the onset of KD, and leads immediately to inflammation of all layers of the artery. The inflammation spreads completely around the artery; as a result, structural components of the artery undergo intense damage; the artery then begins to dilate. KD arteritis is characterized by granulomatous inflammation that consists of severe accumulation of monocytes/macrophages. Aberrant activation of monocytes/macrophages is thought to be involved in the formation of vascular lesions (37).

A 2023 study by Cao et al. (38) analyzed the PBMC of 82 Kawasaki Disease (KD) patients through flow cytometry and bulk RNAseq, with 6 patients further having their PBMC analyzed with scRNAseq. Comprehensive weighted gene co-expression network analysis was used to analyze bulk sequencing results for gene upregulation in monocytes that were strongly correlated to KD. GO analysis of these DEG found that Kawasaki patients have genes correlated with the proliferation of PBMC significantly upregulated. As KD causes severe inflammation to the point of causing vascular damage before typically self-resolving, it is logical for the monocyte population to rapidly expand in this condition.

Upon flow cytometry, a significant increase in the population of B cells was discovered, with the populations of CD4+ and CD8+ T cells, as well as NK cells, being significantly decreased. Notably, the ratio of CD4+/CD8+ T cells was higher in the KD group than in the control group.

scRNAseq revealed that 30% of the total cell population of KD PBMC was composed of CD4 T cells and B cells. As immune landscape changes suggested a link between CD4+ T cells and KD, the T cells were extracted from the dataset, and reanalyzed, demonstrating that 83% of T cells were naive, 13% were Treg cells, and 4% were T helper 2 cells; the majority of cells differentiating into Treg and Th2 suggests they play a major role in the vascular damage the inflammation of this disease causes. These three types of cells were also found to be enriched in the COVID signaling pathway, with Treg and T helper 2 cells primarily associated with viral or bacterial infection and cardiomyopathy pathways. Unfortunately, the small size of the cohort analyzed by scRNAseq didn’t allow authors to make some strong conclusions and also a comparison of flow cytometry data with scRNAseq can be helpful to draw the most optimal way of clusterization of KD patients. Obviously, the sample needs to be expanded to identify any patterns within patients and to find links with prognosis and therapy.

### 3.3 Systemic lupus erythematosus

Systemic lupus erythematosus (SLE) is a quintessential autoimmune disease, marked by recurring episodes and periods of remission, with the potential to cause extensive damage to multiple organ systems and tissues. A defining characteristic of SLE is the production of autoantibodies that target self-antigens, leading to the creation of immune complexes. These complexes accumulate within blood vessels, triggering intense inflammatory reactions that can lead to the dysfunction of various organ systems. Recent medical advances have shed light on the etiology of SLE, with increasing recognition of dysregulated immune mechanisms involving both the adaptive and innate immune compartments (39). Perez et al. (40) analyzed the PBMC of 162 patients with systemic lupus erythematosus using bulk sequencing and single cell transcriptomics. They found that lupus patients had CD4 T cell lymphopenia, a decrease in naïve CD4+ T cells, clonal expansion of cytotoxic GZMH+ T cells, and a limited presence of CD8+ T cells, yet not CD4+ T cells. Notably, patients receiving oral steroids had an increase in CD8+ T cells, and those receiving immunosupressor azathioprine had a decrease in NK cells, despite this population not being different from healthy controls.

### 3.4 Rheumatoid arthritis

Rheumatoid arthritis (RA) is a chronic destructive inflammatory synovitis, accompanied by wider clinical sequelae including comorbidities particularly affecting systemic bone, vasculature and metabolic function, and cognition (41).

Lewis et al. (42) analyzed 90 patients with early RA that were naive to therapy through scRNA of synovial fluid, with the sample size comprising 87 after quality control (QC); analysis of the whole peripheral blood of 67 of these patients was also analyzed. They found that synovial fluid analysis could be reliably used to differentiate between RA pathotypes, namely lympho-myeloid, pauci-immune fibroid, and diffuse-myeloid based on their immunophenotype profiles; they were also found to strongly correlate with results obtained from more invasive methods, such as biopsy of synovial membrane. Unfortunately, in this study, analysis of peripheral blood was found to be less robust, with only 8 differentially expressed transcripts between pathotypes, as compared to 3,000 in synovial fluid, allowing observation of just two RA pathotypes: diffuse-myeloid and pauci-immune fibroid. Additionally, synovial fluid could be reliably used to track response to disease-modifying antirheumatic drug (DMARD) therapy, whereas PBMC analysis was not found to have robust enough differences to be used for tracking progression. Despite this, PBMC analysis was found to be potentially beneficial as a non-invasive diagnostic tool, albeit one that is less specific than that of synovial fluid.

Hedman et al. (43) analyzed the PBMC of 90 RA patients using flow cytometry and glucocorticoid signature analysis. In both early and established RA patient immunophenotypes, classical and non-classical monocytes, as well as T helper 1 and 2 cells were elevated, while NK and B cells were significantly decreased in early and established RA, respectively. Notably, an elevation in the level of B cells was noted in patients with early RA, especially B memory cells. Glucocorticoid but not MTX treatment was found to strongly correlate with restoration of monocyte population proportions. Treatment with MTX was found to reduce memory and plasma B cells and CD4+ T cells (Th1 and Th17). TNF inhibitor (TNFi) treatment in patients with established RA significantly increased mature B cell levels, with minimal effect on other cell types. Authors noticed that a decrease in mature B cells were significantly associated with RA compared to healthy controls, suggesting that changes in these populations in patients during treatment may be associated with the treatment outcome.

Decreases in mature B cells and increases in monocyte populations were significantly associated with RA across the board, so it was concluded that restoration of these populations to levels more comparable with healthy controls was indicative of treatment outcome. Based on this hypothesis, the team created a prediction algorithm to determine treatment outcomes prior to therapy. They found good success in predicting outcomes for MTX response, with less precision with patient response to TNFi. This is likely due to the significantly smaller sample size of patients treated with TNFi (37 as compared to 53).

While further research is needed in this area, with significantly larger populations, this is an area where immunophenotype analysis has the potential to have extremely significant influence in the sphere of treatment outcomes.

Kubo et al. (44) performed peripheral blood immunophenotyping of two large cohorts of RA patients. The first cohort, the discovery cohort, was initially composed of 533 immunophenotyped patients, then, after 24 weeks of DMARD treatments, 290 of the original cohort were once again phenotyped. The second cohort, the validation cohort, initially comprised 206 patients. After phenotyping, 21 patients with low disease activity were excluded; the remaining 185 patients were then treated with DMARDs and periodically checked in on in a 26 week period.

Analysis found considerable heterogeneity between RA patients, with clusters not fully corresponding to typical criteria based on best practice clinical syndromes or serum markers. Notably, certain groups of RA patients were found to have immunophenotypic patterns comparable to those of healthy controls. Patients were clustered into 5 distinct groups – peripheral blood cell abundance phenotypes- little difference (PCAP-LD), peripheral blood cell abundance phenotypes- slightly difference (PCAP-SD), peripheral blood cell abundance phenotypes- T cell and B cell activation (PCAP-TB), peripheral blood cell abundance phenotypes- TEMRA CD4 activation (PCAP-T4), and peripheral blood cell abundance phenotypes- TEMRA CD4 and Th1 activation (PCAP-T4T1).

Immunophenotyping results were affected by many factors, including age (PCAP-LD patients were younger, though a similar pattern of differences were observed in controls based on age), glucocorticoid, and MTX use.

This study found that immunophenotype at treatment may dictate prognosis results. JAK inhibitors, though their use was relatively limited across the patient cohort, were found to be linked to increased efficacy of therapy. IL-6 inhibitors were found to be most effective in PCAP-LD patients, while TNF inhibition was best in PCAP-T4, and CTLA4-Ig in PCAP-T4T1. Groups treated with “expected” DMARDs outperformed those that were not by 15.3% (39.9% *vs* 24.6% achieved remission), with patients in the low activity group outperforming by 20.6% (80.8% *vs* 60.2% remission).

## 4 Immunotypes of cancer

In cancer, researchers have also begun to step towards the concept of individual populations and towards the development of immune signatures in peripheral blood that have prognostic or predictive value. One of the first attempts was the work of Bhutani et al. on patients with multiple myeloma (45). Immunotyping results showed significant changes in the peripheral compartment of mature NK cells in minimal residual disease (MRD) neg and MRDpos patients. MRDpos patients had a reduced proportion of circulating NK cells compared with the MRDneg counterpart. In the MRDpos group, NK cells more frequently expressed the activating receptor KIR2DS4 and less frequently expressed the inhibitory receptor NKG2A. This suggests that NK cells induced in the periphery in patients with MRDpos retain the activating receptor KIR2DS4 activation capacity. In addition, patients with MRDpos showed a deficiency of peripheral NKG2A+ NK-T-like cells and KIR3DL1+ T cells compared to the MRDneg group. These results indicate that MRD status differs in the immunotype of mature NK, NK-T-like, and T lymphocytes in peripheral blood, which was hypothesized to be used as a prognostic test system.

Shen et al. (46) analyzed the peripheral blood immune status of 188 melanoma patients receiving immune checkpoint blockade (ICB) therapy (either antibodies to PD-1 (n=76, 40%), antibodies to CTLA-4 (n=13, 7%), or both in combination (n=99, 53%)). The authors applied cluster analysis (survClust) to stratify patients based on a multivariate model of 78 flow cytometry parameters. Careful cross validation analysis identified 3 groups of patients with different expression patterns of immune markers. The first immunotype was unequivocally characterized by high expression of LAG-3 (lymphocyte-activation gene 3) in multiple T cell populations, the most representative of which were LAG-3+CD8+ T cells, as a consequence of which the authors named the immunotype LAG+. The LAG+ immunotype was represented by 17.0% (23 of 136) of patients and was partially defined by the presence of Ki67-LAG-3+CD8+ T cells. The second immunotype (LAG-) reflected 65.4% (89/136) of the population and was defined by low numbers of LAG-3+ T cells and low levels of other associated markers in T cells. The third immunotype had a high proportion of LAG-3+ T cells with concurrent high numbers of proliferating Ki67+ CD8+ T cells and T cells expressing TIM-3 and ICOS. The authors named this type the proliferative (PRO) immunotype, and it accounted for 17.6% (24 of 136) of patients.

Melanoma patients with LAG+ immunotype had worse outcomes after ICB with a median survival of 22.2 months compared to 75.8 months for patients with LAG- immunotype. An independent cohort of 94 urothelial carcinoma patients receiving ICB (included also in this study) in whom the LAG+ immunotype was significantly associated with response, survival, and progression-free survival was used for validation. Multivariate Cox regression analysis and stratified analysis also show that LAG+ immunotype is an independent indicator of outcome compared to known clinical prognostic markers and previously described biomarkers. Thus, the authors concluded that the LAG+ immunotype identifies patients who are significantly less likely to benefit from ICB. In the context of this study, it is interesting that Lag-3 is a checkpoint inhibitor molecule, including determining the exhausted phenotype of immune cells (47). Thus, we can assume that the LAG+ cluster is a group enriched with lymphocytes with suppressed effector function. This, among other things, may explain the negative effect on survival - the presence of an additional suppressor mechanism besides PD1 enhances suppression of immune system activity and creates conditions for bypassing immune activation due to the use of PD-1 inhibitors. This study provide an example of potentially useful determination of immunotypes during treatment in order to timely correct therapy, i.e. it has been shown that combination therapy aimed at blocking both PD-1 and Lag-3 can be used for this group (48).

In an analysis of a patient cohort of 804 patients with metastatic castration-resistant prostate cancer (mCRPC) treated with dendritic cell vaccines and chemotherapy, Hensler et al. (49) identified two major clusters of patients. Cluster 1, a high inflammation cluster, was significantly enriched with 68 immune-related genes compared to cluster 2, a low inflammation cluster (CD3E, CD8A, IL2, STAT4, GATA3, CD28, ICOS, Lag-3, CTLA4, FOXP3 and others). This observation was confirmed by analyzing the functional activity of selected genes, which revealed a significant association between DEGs, in particular the positive regulation of adaptive immune response as well as cytotoxic T and NK cell immunity. In both study groups, the high inflammation cluster was associated with longer OS (p<0.001) compared to the low inflammation cluster.

Non small-cell lung cancers (NSCLCs), similar to mCRPCs, had longer OS in the high inflammatory cluster, with 44 genes associated with T cell activation were significantly represented in two arms of the study. Autologous DC-based vaccine (DCVAC) treatment conferred a significant OS advantage to patients with high expression of genes associated with B cells, CD8A+ T cells, and DCs.

In contrast, epithelial ovarian cancer (EOC) had better OS in the low inflammatory cluster, though only when treated with DCVAC. A negative prognosis was found to be associated with 5 genes in DCVAC patients, namely CD3E, CD4, forkhead box P3 (FOXP3), granzyme A (GZMA), granzyme B (GZMB), HLA-DOB, and interleukin 4 (IL4). A high frequency of regulatory T cells in peripheral blood of EOC patients was associated with poor response to DCVAC.

Chauchan et al. (50) examined the PBMC of 104 patients with HER2 negative breast cancer using flow cytometry, with a further 63 of these patients also having biopsies analyzed by the same method. Breast cancer patients were found to have significantly higher percentages of monocytes, and lower percentages of pDCs and CD4+ T cells in PBMC. Patients were then separated by breast cancer type – hormone receptor positive (HR+) and triple negative (HR-) - and it was found that HR positive patients had significantly higher levels of NK cells. Furthermore, triple negative patients had higher neutrophil and lymphocyte counts, as derived from whole blood analysis.

Upon analysis of treated and untreated breast cancer patients to determine whether it was treatment affecting the patients’ immunophenotypes, it was found that there were no significant differences between the phenotypes of untreated patients and healthy donors, while patients who previously received chemotherapy or CDKi had a lower percentage of pDC and CD4+ T cells, and a higher percentage of monocytes. When comparing treated and untreated patients, patients who underwent treatment also had lowered lymphocyte counts.

Notably, there were no differences found between the phenotypes of patients who underwent treatment previously, and those currently undergoing treatment, suggesting that these changes may be permanent.

Patients were found to have significantly higher EM CD4+ T cells and lower CD4+ T cells than healthy donors, with this effect being more pronounced in patients who had gone through treatment. Additionally, treated patients had significantly higher levels of naive B cells, and lowered levels of memory B cells, with this trend further influenced by therapy – these patients had more naive but fewer non switched memory B cells. M2 monocyte levels were lowered in patients. Despite having comparable levels of myeloid-derived suppressor cells (MDSc) to healthy control across all groups, treated patients were found to have a significantly higher MDSC to T cells ratio, with therapy reducing this ratio. MDSC to T cell ratio was significantly higher in BC patients compared to HD. Patients had reduced CD16− mDC levels, and slightly higher levels of the highly cytotoxic CD57+ NK cells.

T cell phenotypic markers suggested that patients had higher levels of T helper 2 and 17 cells. Further analysis found that the CD4+ T cells and T regulatory cells from patients expressed high levels of immune checkpoint receptors, with Tregs further expressing higher levels of CD39.

Further analysis was done to compare the relevance of PBMC immunophenotypes to those within the tumor itself on the 63 patients who had biopsies taken in a similar timeframe to the PBMC. It was found that CD4+ T cell, NK cell, and Mo-MDSCs proportions in PBMCs correlated to their proportions within the tumor.

None of the major subsets of immune cells were found to be significantly associated with therapeutic outcome, other than pDCs, which were correlated to poor outcome in the placebo-chemo arm of the experiment. CD4+ TEMRA T cells were positively associated with outcome in the placebo-chemo arm, while CD56bright NK and CD8+ naive T cells were associated with treatment resistance. When it came to the atezo-chemo arm, patients with higher levels of Granzyme-B+ CD8+ T cells and switched memory B cells correlated with positive outcomes, while increased numbers of PD1+ CD4+ T cells and increased proportions of a naive phenotype in B cells were associated with inferior outcome.

The most comprehensive study of peripheral blood immunotypes in cancer is the work by Dykanov et al. (51), summarizing data from 408 healthy donors and 442 cancer patients, ages 16 to 98 years (total n = 850). This cohort contained samples from patients with 84 different solid tumors and 7 types of therapy. Frequencies of up to 650 cell types and activation states were measured for each sample, and uniform multiple approximation and projection (UMAP) was used to display these characteristics in two dimensions. The authors noted that patients with similar diagnoses did not form distinguishable groups, nor did patients with similar lines of therapy. Conversely, healthy donors and cancer patients formed separate groups. Patients in different age groups also tended to cluster together, which is consistent with both studies targeting healthy donors and the fact that healthy donors tend to be younger than patients with solid tumors. Thus, differences in immune profiles between individuals in this pansolid cohort may be explained by the presence or absence of cancer, regardless of tumor type or treatment.

Five immunotypes (G1-G5) were identified using this approach. In group G1, a high frequency of naive CD4+ T cells, naive CD8+ T cells and naive B cells was detected. Group G2 revealed a higher percentage of differentiated CD4+ central and transient memory T cells, as well as CD39+ Treg. G3 showed an increased frequency of mature NK cells as well as CD8+ transient memory and PD-1+ TIGIT+ CD8+ T cells. G4 was enriched with NKT cells as well as terminally differentiated effector memory CD45RA+ (TEMRA) and CD45RA- (TEM) both CD4+ and CD8+ T cells. Finally, G5 was enriched with classical monocytes, HLA-DRlow monocytes and neutrophils and contained fewer lymphocytes. The presence or absence of a cancer diagnosis (healthy or cancer) and the age of the patients were not evenly distributed between immunotypes. Importantly, immunotypes G4 and G5, enriched with terminally differentiated CD8+ T cells and classical monocytes, respectively, contained very few healthy donors. Conversely, the G1 immunotype, with the highest percentage of naive T and B lymphocytes, contained the highest proportion of healthy donors. The most frequently presented diagnoses in our internal cohort contained similar ranges of immunotypes, suggesting that cancer type was not a major factor in the distribution of immunotypes.

In addition, the authors evaluated the predictive role of immunotypes in different tumors. Interestingly, breast cancer patients with partial complete response to neoadjuvant chemotherapy were more likely to belong to the G5 immunotype than patients with residual disease (RD). Patients with partial response to neoadjuvant chemotherapy had a significantly higher G5 signature and a significantly lower G1 signature than RD patients. The binary response stratification ROC-AUCs for G5 and G1 response were 0.79 and 0.25, respectively, suggesting that immunotypic characteristics are sensitive to systemic changes associated with response. G3 and G4 immunotypes tended to develop a response in the same direction as G5, while G2 is close to G1, indicating a broader shift in immune system composition predicting effective responses to chemotherapy.

To assess the predictive role of identified immunotypes on immunotherapy treatment, the authors analyzed two cohorts: 32 HNSCC treated PD-L1 inhibitors with anti-PDL1 durvalumab and 35 HNSCC patients (HNSCC-Nivo cohort) receiving first-line anti-PD-1 nivolumab alone or nivolumab in combination with the indolamine-2,3-dioxygenase-1 inhibitor BMS-986205 (IDOi). In the first cohort analysis, no statistically significant association of a specific immunotype with treatment response could be identified, but responders tended to fall into the G4 cluster. When comparing the predictive power of the G4 signature with the measurement of PD-L1 expression in tumors by RNA-seq in patients from this cohort, the G4 signature was shown to be significantly superior to the assessment of PD-L1 expression in tissues when stratifying pre-treatment response.

In the second case, responders were most often of the G2 immunotype, which allowed prognostically distinguishing responders from non-responders with an accuracy of 76%. All patients with a G2 profile responded to nivolumab. In this context, the G2 signature demonstrated potential utility as a prognostic biomarker for treatment of advanced HNSCC with nivolumab.

## 5 Immunotypes of other diseases

The concept of immunotypes was actively developed during the COVID-19 pandemic. A team of authors from the University of Pennsylvania (52) encountered a number of problems when examining the effect of individual subpopulations on disease severity when analyzing the immune response to COVID-19. First, there was significant heterogeneity between patients for each immune parameter associated with the assessment of disease severity. Second, these binary comparisons (e.g., one immune subpopulation versus one clinical feature) did not allow for full utilization of multivariate information in this dataset. This forced the use of feature-weighted kernel density with UMAP visualization, which allowed the discovery of similar patterns of immune system activation, converging on the order of 200 parameters. Using this approach, the researchers identified three immunotypes: (i) Immunotype 1 was associated with disease severity and manifested by high CD4- T cell activation, lack of circulating follicular helper cells, activated CD8-”EMRA”, hyperactivated or depleted CD8- T cells. (ii) Immunotype 2 was characterized by reduced activation of CD4+ T cells, Tbet+ effector CD4- and CD8+ T cells, proliferating memory B cells, and was not associated with disease severity. (iii) A third immunotype was also identified, which was negatively correlated with disease severity and had no obvious activated T and B cell responses. Immunotype 3 was defined as the intersection of the bottom 50% of five different flow parameters: PB as percentage of B cells, KI67+ as percentage of non-naïve CD4+ T cells, KI67+ as percentage of non-naïve CD8+ T cells, HLA-DR+CD38+ as percentage of non-naïve CD4+ T cells, and HLA-DR+CD38+ as percentage of non-naïve CD8+ T cells.

As for the general differences from healthy donors, the patients had increase in the CD8+ CD45RA−CD27−CCR7+ EM and CD45RA+CD27−CCR7− EMRA populations, and a decrease in CD45RA−CD27+CCR7− EM cells. A significant increase in KI67+ and HLA-DR+CD38+ non-naïve CD8+ T cells in COVID-19 patients relative to HDs or RDs which indicates standard response to the viral infection was also observed (53).

Thus, this study allows us to separate the group at increased risk of complications in covid-19 based on the determination of the immunotype, which can significantly facilitate the prediction of the effectiveness of therapy and personalize the approach to disease management.

In a study by Bodinier et al. (54), two immunotypes in critically ill patients (septic patients undergoing major surgery or severe trauma) with prognostic significance were identified. Immunotype 1, which had a negative prognosis, was characterized by elevated levels of IL6 and IL10 in plasma, a significant increase in the number of immature neutrophils (up to 80% during the first week after trauma), and consistently low levels of mHLA-DR. These patterns suggest a more pronounced dysregulation of the immune system in patients with immunotype 1 immediately after injury. Interestingly, T lymphocyte counts in both immunotypes were comparable and within the range observed in healthy volunteers. Although Immunotype 2 was also statistically significantly different from healthy donors in terms of IL6, IL10, and neutrophil counts, but had milder changes with a slower immune response, its profile was closer to those of Immunotype 1, as well as in response to LPS and Staphylococcal Enterotoxin B (SEB) stimulation. This fact indicates a significant dysregulation of the immune system in Immunotype 1, which appears to lead to a worsening of the course of the disease.

In another study, researchers performed a deep immunoprofiling assay of patients with chronic kidney disease (CDK) and healthy donors. It was found that three cell clusters associated with CD56dim NK cells and B cells, respectively, and one cluster corresponded to a combination of CD56bright NK cells; a subset of monocytes were decreased in CKD. To test the potential of immunotypes as diagnostic markers, authors constructed a random regression model based on the 19 cellular and soluble parameters identified in this study to distinguish patients with CKD from non-CKD controls. Using 19 immune variables, an overall area under the ROC curve (AUC) of 0.917 was obtained to identify a patient with CKD, with the most important parameter being the proportion of CD38+ monocytes. In addition, slightly worse but still satisfactory results were obtained in identifying a mild degree of CKD compared with the control group, with an AUC of 0.889. These results reveal promising possibilities for early diagnosis of CKD using specific immunotypes. (55).

## 6 Discussion

Different pathologies may have different key populations selected, and their number and the number of directly detected immunotypes may vary. In addition, the immunotype identified is not always clearly associated with the prognosis of the disease or the effect of therapy. Many of the studies presented above simply compare healthy donors and patients with a specific disease, finding differences in immunotypes. However, it has been shown that the inflammatory process has similar mechanics in various diseases (56), suggesting a possible similarity in immunotypes between diseases.

As we described above, authors apply different methods such as flow cytometry (and different multicolor panels), scRNAseq and bulk RNA seq, as well as different deconvolution models of RNAseq data, different clustering methods and visualizations (such as PCA analysis, t-SNE, UMAP). Previously, it was shown that RNAseq and flow cytometry demonstrate partial correlation and differ between immune subsets (57). Despite this fact, in the study of Dyikanov et al, immunotypes defined by RNAseq and flow cytometry were very close in their content and biological meaning. It seems logical that standardization of using methods, panels and equipment probably may allow to define more consistent immunotypes between diseases of the same nature.

**Figure 2:**
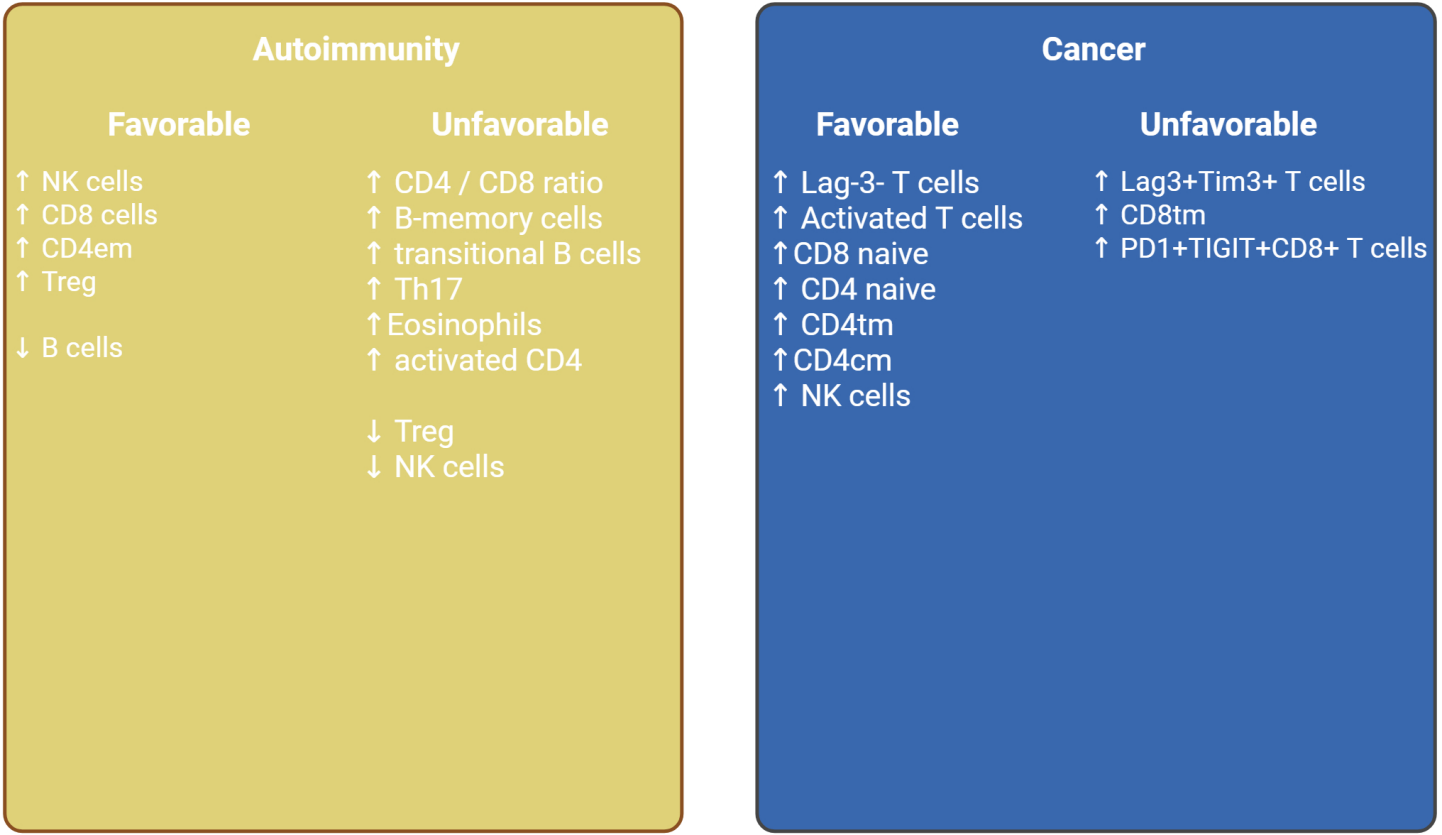
Key cell populations based on reviewed studies stratified by favorable and unfavorable impact in context of autoimmunity and cancer.

It is important to note, that despite such heterogeneity in methods and diseases, in all subsets of the studied individuals, clusters enriched in naive lymphocytes, effector immune cells, and clusters with a pronounced predominance of immunosuppressed populations are distinguished. Therefore, the assessment of immunotype is reduced to the evaluation of the ratio of naive and activated, suppressor and effector links. The main cell types that allow for the stratification of immunotypes and the predictive role of the immunotypes identified in various conditions are shown in **Table 2**. The purpose of this review was primarily to summarize the available data on subpopulations enriched in various diseases and causing favorable and unfavorable prognosis. This attempt to summarize immunotype studies and identify key populations is shown in **Figure 2**. As we can see based on this figure, for both cancer and autoimmune diseases, the populations defining a particular prognosis are close to each other in biological terms and essentially represent one or another immunotype with activation or exhaustion. Based on this fact, we can tailor therapy to the specific characteristics of an immunotype. For example, for an immunotype with a pronounced pro-tumor profile in cancer, we can use immunotherapy, taking into account its unique features (such as the expression levels of specific molecules like CTLA-4, PD-1, Lag-3, etc. or need in monocytes reprogramming). At the same time, it seems interesting to conduct a meta-analysis of the effectiveness of existing therapies for each immunotype in order to personalize existing options, as verification of the selection of therapy based on the molecular characteristics of the immunotype requires clinical trials.

**Table 2:** - Table summarizing the immunotypes identified in different conditions and diseases.

Disease	Number of patients and controls	Methods	Number of immunotypes	Discoveries	Source
Healthy population	398 individuals	Flow or mass cytometry, functional response measurements	3	Immune variance correlated with age, gender, and CMV seropositivity CMV seropositive patients had profiles close to the aging phenotype Samples with marked imbalances in monocytes, T lymphocytes, or NK cells did not cluster separately No detailed description of specific immunotypes of healthy donors Euclidean distance between points representing twins smaller than between unrelated donors, pointing to genetic influence	(26)
	210 twins (105 pairs)	Flow and mass cytometry, immune cell signaling, serum protein quantification, hemagglutination inhibition assays, CMV serology	none	Immune system variation is largely non-heritable Immune system variance increases with patient age	(29)
	326 individuals	Hematology analysis, flow cytometry, CMV and EBV seropositivity analysis	9 1 Increased: CD8 True Naive T cells Decreased: CD4+ Treg, CD4+ CD95+, CD8+ CD95+, CD4+ Tcm, CD8+ Tcm 2 Increased: Classical monocytes, CD4+ HLA-DR+, CD4+ Tcm, CD8+ Tem Decreased: CD8+ True naive 3 Increased: CD8+ True naive, classical monocytes, CD4+ Tscm Decreased: CD4+ CD95+ 4 Increased CD8+ True naive, CD4+ HLA-DR+, CD4+ Tcm Decreased: CD4+ Treg 5 Increased: CD4+ Tscm Decreased: CD8+ True naive, classical monocytes, CD4+ HLA-DR+, CD4+ Tcm, CD8+ Tcm 6 Increased: CD8+ True naive, CD4+ Tscm, CD8+ Tscm Decreased: CD4+ HLA-DR+, CD4+ Tcm 7 Increased: CD4+ Treg, CD4+ CD95+, CD8+ CD95+, CD4+ HLA-DR+, CD4+ Tcm, CD8+ Tem Decreased: CD8+ True naive 8 Increased: CD19+ CD95+ v CD8+ True naive, CD4+ Tscm, CD8+ Tscm 9 Increased: CD8+ CD95+, CD8+ Tscm, CD4+ Tcm, CD19+ CD95+ Decreased: CD8+ True naive	Immunotype is defined by age; younger individuals have higher levels of CD4 and CD8 true naive T cells Older individuals show signs of immune system remodeling Immunotype 5 and 8 were largely defined by CMV seropositivity	(30)
	305 individuals from the above study	Serum antibodies and vaccine response profiles, CMV seropositivity	see above	CMV seropositivity had no effect on vaccine response Immunotype 1 and 6 had increased odds of belonging to a higher quartile of triple vaccine response Immunotype 8 had significantly lower vaccine sensitivity	(31)
Diabetes	826 patients	AAB measurements, flow cytometry, CBC, HbA1c, and blood glucose measurement, CMV status, genotyping of T1D risk loci	? No direct immunotypes, phenotype trajectories of disease in terms of correlation to aging. T1D Increased: CD4+ T cells Decreased: B cells and CD8+ T cells; naive -> memory cells in adaptive immune system	A model for predicting DM1 while correcting for the age-related changes was developed Initially similar to healthy donors, then deviate over the first 30 years of life Direct comparison demonstrated time/age-linked trajectories of the phenotypes, pointing to distinct, age-related changes in DM1 patients Patients showed accelerated immune aging, unrelated to CMV status, glycated hemoglobin levels, and genetic risk of developing DM1	(34)
	12 patients	Mass cytometry	2 T1D Increased: activated Tregs, activated CD4+ T cells activated CD8+ T cells, CD4+CD8+ T cells, Th1 T cells, Th17 T cells, transitional B cells, memory resting B cells, memory B cells, immature eosinophils Healthy Increased: Th2 T cells, IgD, galectin-10+ eosinophils	spanning tree of major cell populations present in the blood of twelve patients with T1D and twelve healthy subjects showed differences in immune system balance Absence of galectin-10hi eosinophilic subgroup in individuals with T1D compared with healthy controls.	(58)
	46 patients	scRNAseq, SNP-array genotyping, FACS	2 T1D Increased: Mo/cDC cells, naive and effector T cells, CD8+ naive T cells, C-monocytes, pDC, CD4+ and CD8+/Treg cell ratio Healthy Increased: lymphoid cells, CD4+ EM cells, Treg cells	TMZ score HLA risk types for T1d had no effect on clusterization, though B cell DEGs showed the highest correlation with heritability, largely driven by HLA locus risks. TMZ score was T1DM metagene z-score, which was assigned in such a way as to allow for the differentiation between patient subtypes, as well as between patients and controls. 29/31 controls were in the low-response group, as were 14/46 T1D patients, suggesting they may have a milder systemic immune response. The intermediate and high response groups were 33/35 T1D cases, with the high response group including a single control patient, who was diagnosed with T1D 4 years after blood was collected for the research. Verapamil, abatacept, and rituximab decreased TMZ score, teplizumab did not.	(35)
	29 patients	Mass cytometry	3 ≥2 autoantibodies Increased: NK cells and γδ T cells Decreased: CD4+ and CD8+ T cells GADA Increased: NK and CD8+ T cells Decreased: B cells IAA Increased: CD39 levels on CD4+ T cells	Different T1D phenotypes can be predicted by immunotyping of peripheral blood	(36)
Kawasaki disease	82 patients	Flow cytometry, bulkRNAseq	2 Kawasaki Increased: B cells, ratio of CD4+/CD8+ T cells Healthy Increased: CD4+ and CD8+ T cells, NK cells	30% of the total cell population of KD PBMC was composed of CD4 T cells and B cells 3% of T cells were naive, 13% were Treg cells, and 4% were T helper 2 cells	(38)
Systemic lupus erythematosus	162 patients	Bulk sequencing, single cell transcriptomics	2 Lupus Increased: CD4+ T cells, GZMH+ T cells Healthy Increased: CD4+ T cells, CD8+ T cells	Oral steroids increased CD8 T cells Azathioprine decreased NK cells	(40)
Rheumatoid Arthritis	90 patients	scRNAseq	2 Rheumatoid Arthritis Increased: IFI27, LY6E, ISG15, USP18, OASL, RSAD2, IFI44L, C4BPA	Separation and prediction of 4 RA pathotypes by DEGs in the whole blood and tissue.	(42)
	90 patients	Flow cytometry, glucocorticoid signature analysis	2 Early RA Increased: classical and non-classical monocytes, T helper 1 and 2 cells, B cells, B memory cells Decreased: NK cells, mature B cells Established RA Increased: classical and non-classical monocytes, T helper 1 and 2 cells Decreased: B cells	Glucocorticoid treatment restored monocyte population proportions MTX treatment reduced B cell (memory and plasma) and CD4 T cells (Th1 and Th17), with limited effects on monocyte populations Both treatments reversed B cell levels to control-comparable levels	(43)
	739 patients total	Flow cytometry	5 PCAP-LD little difference from healthy controls PCAP-SD Decreased: TC1 cells PCAP-TB Increased: CD4+Tcells, CD8+T cells, plasmablasts PCAP-T4 Increased: CD4+TEMRA, effector memory T cells PCAP-T4T1 Increased: CD4+TEMRA, Th1 cells	Immunophenotype was affected by age, glucocorticoid and MTX use Immunophenotype at treatment may dictate prognosis results IL-6 inhibitors were found to be most effective in PCAP-LD patients, while TNF inhibition was best in PCAP-T4, and CTLA4-Ig in PCAP-T4T1. Groups treated with “expected” DMARDs outperformed those that were not by 15.3% (39.9% *vs* 24.6% achieved remission), with patients in the low activity group outperforming by 20.6% (80.8% *vs* 60.2% remission).	(44)
Multiple Myeloma	46 patients	Flow cytometry	2 MRDneg Increased: NK cells, NKG2A+ NK-T-like cells, KIR3DL1+ T cells MRDpos	Proportion of NK-T-like cells increased with IMiD treatment, over time Mature NK cells went through loss of effector function with IMiD treatment T cells acquired an early PD1-independent anergic state with IMiD treatment MRDpos patients gained increased NK cells, NKG2A+ NK-T-like cells, KIR3DL1+ T cells, and NKG2A+ T cell during lenalidomide maintenance therapy MRDpos has increased KIR2DS4+NK cells during lenalidomide maintenance therapy	(45)
Melanoma and Urothelial cancer	188 ICB-treated melanoma patients 94 ICB-treated urothelial carcinoma patients	Flow cytometry	3 LAG+ Increased: LAG3+ T cells LAG- Decreased: LAG3, TIM3+ T cells, ICOS+ T cells, Ki67+ T cells PRO Increased: LAG3+ T cells, TIM3+ T cells, ICOS+ T cells	**Melanoma** LAG- had best survival LAG+ had worst outcomes on anti-PD-1 monotherapy Immunotype did not have significant effect on combination therapy of ipilimumab and nivolumab LAG- higher PFS LAG+ is independent of known clinical prognostic factors **Urothelial cancer** LDH was significantly associated with immunotype LAG+ phenotype significant PRO worse survival LAG+ associated with worst survival, no treatment response LAG- Best OS and PFS LAG+ and PRO poor prognosis not associated with previously-defined markers	(46)
mCRPC, NSCLC, EOC	804	Flow cytometry	2 High Inflammatory Cluster Increased: 68 inflammatory genes in McRPC, 61 in NSCLC, 68 in EOC Low Inflammatory Cluster	**mCRPC** High Inflammatory Cluster associated with longer OS 43 genes associated with adaptive immunity and T cell activation were significantly represented in two arms of the study No advantage DCVAC in either cluster DCVAC treatment conferred a significant OS advantage to patients with high expression of CD8A **NSCLC** High Inflammatory Cluster associated with longer OS 44 genes associated with T cell activation were significantly represented in two arms of the study No advantage DCVAC in either cluster DCVAC treatment conferred a significant OS advantage to patients with high expression of genes associated with B cells, CD8A T cells, and DCs **EOC** DCVAC patients in the low inflammatory cluster had better OS, though not SOC patients A negative prognosis was found to be associated with 5 genes in DCVAC patients, namely CD3E, CD4, forkhead box P3 (FOXP3), granzyme A (GZMA), granzyme B (GZMB), HLA-DOB, and interleukin 4 (IL4) DCVAC patients in the low inflammatory cluster had significantly better PFS, compared to SOC In high inflammatory patients, no difference High frequency of regulatory T cells in peripheral blood of EOC patients was associated with poor response to DCVAC	(47)
metastatic HER2− BC	102	Mass cytometry, flow cytometry, cytokine analysis	2 Breast Cancer Increased: monocytes, effector memory CD4+ T cells, CD57+ NK cells Healthy Donor Increased: CD4+ T, pDCs, naive CD4+ T cells, M2 monocytes, D16− mDCs,	No significant differences in immune cell proportions between HD and untreated patients Previously treated patients had ^monocytes v CD4+ T cell and pDC No significant differences between currently treated and previously treated patients, other than current patients having lower lymphocyte counts CDK inhibitors v neutrophils BC patients receiving treatments had higher MDSC to T cells ratio T cells from BC patients exhibit activated/exhausted phenotype	(50)
different cancers	442	RNA-seq, flow cytometry	2 Cancer Patients Increased: CX3CR1neg CD8+ TEMRA, monocytes Healthy Donors Increased: naive CD4+ and CD8+ T cells, memory B cells 5 G1 Increased: naive CD4+ T cells, naive CD8+ T cells, and naive B cells G2 Increased: differentiated CD4+ central and transitional memory T cells, CD39+ Tregs G3 Increased: mature NK cells, CD8+ transitional memory, and PD-1+ TIGIT+ CD8+ T cells G4 Increased: NKT cells, effector memory CD45RA+ (TEMRA) and CD45RA− (TEM) of both CD4+ and CD8+ T cells G5 Increased: classical monocytes, HLA-DRlow monocytes, neutrophils, lymphocytes	Patients with similar diagnoses or treatments did not form distinguishable clusters Patients in different age groups tended to cluster as well, consistent with older people being patients Disease state differentiated patients more than age G5 most frequent pCR on NAC G3-progressive immunotype had significantly longer OS in PDAC phase II trial G1 HPV- responded best to nivolumab G2 HPV- responded best to durvalumab	(51)
COVID-19	149	Flow cytometry	3 COVID-19 Increased: Non B/T Cells Decreased: B cell and CD3+ T cell Recovered Donors Healthy Donors 3 1 Increased: CD4- T cell, CD8-”EMRA” circulating follicular helper cells Decreased: CD8- T cells and PBs 2 Increased: CD4+ T cells, Tbet+ effector CD4- and CD8+ T cells, proliferating memory B cells 3 - close to HD	CD8 T cells are lost in larger proportion than CD4 T cells in patients Immunotype 1 associated with severe disease Immunotype 3 inversely correlated with disease severity	(52)
Severe injury, sepsis	339	Flow cytometry	2 1 Increased: IL6 and IL10, immature neutrophils v mHLA-DR 2 Increased: IL6 and IL10, immature neutrophils (less than 1)	Immunotype 1 associated with poor prognosis	(54)
CKD	69	Flow cytometry	2 CKD Healthy Donor Increased: CD56dim NK cells, B cells, CD56bright NK cells, monocytes	CKD Increased: SCF CKD disease severity Increased: RANTES, PIGF1, monocytes, NK cells, B cells; Decreased: PDGF-BB and BDGF	Wu et al., 2022

For studies with multiple cohorts, discoveries for the certain diseases highlighted in bold.

Another important observation is that the proximity of the immunotype to healthy donors generally meant a milder course of the disease and a better prognosis. This observation was noted, in particular, in studies of Mathew et al. (COVID), Bodinier et al. (injuries), Dyikanov et al. (solid cancers), Yong et al. (mCRPC, NSCLC, EOC). Despite the fact that it seems intuitive that an effective immune response means a noticeable activation of defense mechanisms in response to tumor growth or infection, it seems that the adequacy and proportionality of such reactions also play an important role. It can be assumed that with excessive activation, the severity of the disease is also aggravated by the body’s reaction to the massive production of cytokines and tissue damage due to active immune processes.

**Figure 3:**
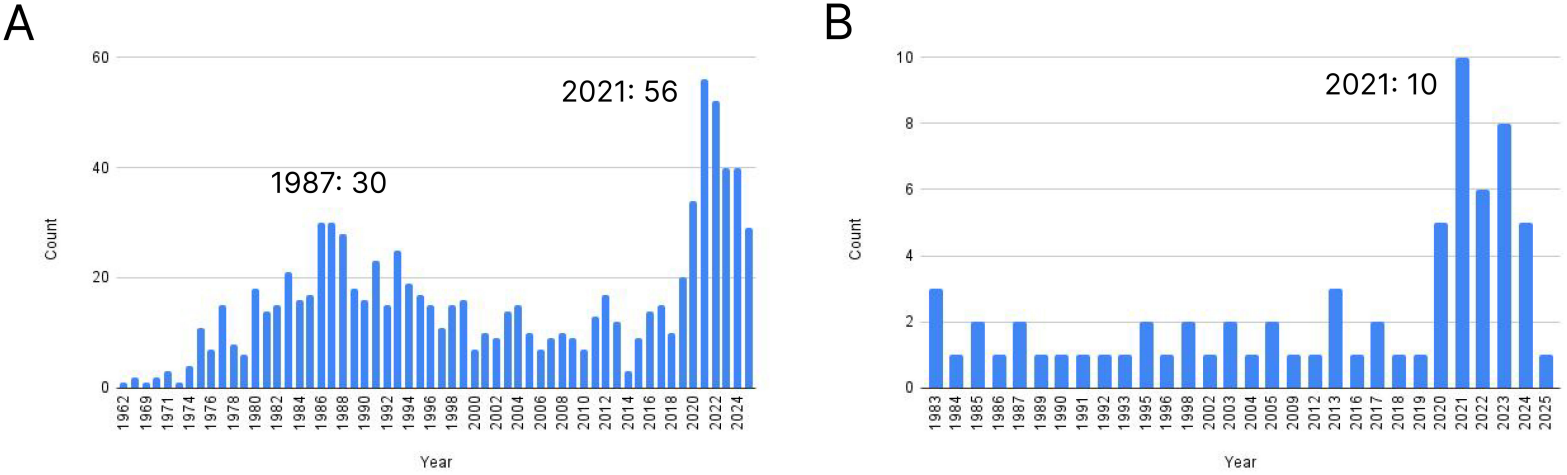
The number of publications in Pubmed system containing the term “immunotype”: **(A)** total number, **(B)** peripheral blood.

Interestingly, the rapid development of the concept of immunotypes started explosively in 2019 and reached a local peak in 2021, although the number of articles has remained low and has even declined over the last two years (**Figure 3**). Notably, the majority of articles are related to the definition of immunotype in tumor tissue as a factor determining tumor pathogenesis and development, with the description of peripheral blood immunotypes accounting for a rather small proportion of studies (a maximum 10 studies in 2021). It should be noted that the previous peak of publication activity (1987: 30) with the using of the term “immunotype” was associated with the study of microorganisms such as Pseudomonas aeruginosa and Neisseria meningitidis, and are not related to the definition of donor clusters based on their ratio between immune populations.

Thus, it should be noted that the use of the term “immunotype” is not widespread, which significantly complicates the search for works related to the clustering of study cohorts by the type of their immune system balance. Despite the intuitive logic and comprehensibility of the term, many researchers either estimate the impact of individual populations under the term “immunotype” or use such constructs as “immunophenotypic heterogeneity”, or “diversity of immune landscape”, which, in our opinion, can be interpreted somewhat broader than the described approaches to clustering (59–61).

Currently, immunotyping of the healthy population is primarily used to predict the effect of vaccination, as well as to study age-related changes in the immune system (28, 30). The latter task is of particular importance, since the age-specific context of the proposed sample must be taken into account when studying the pathogenesis of various diseases and the role of immunity in it, and building predictive models. The foregoing studies show that, although CMV seropositivity plays a role in the formation of immunotype, age still plays a decisive role. If age is not taken into account, various false positives are possible, significantly reducing the accuracy of the models (34).

The concept of immunotypes in cancer has also been widely used. In this case, immune composition serves to predict the outcome of the disease and to predict the efficacy of immunotherapy (51). Of course, researchers are most interested in the immunotype of the tumor itself, but since peripheral blood is a source of immunocompetent cell recruitment, evaluation of its structure also plays an important role.

In the context of autoimmune diseases, researchers are currently limited to examining the differences between cohorts of healthy donors and patients, as well as assessing the genetic predisposition to certain forms of immune imbalance in autoimmunity. This approach undoubtedly plays a great role in studying possible mechanisms of pathogenesis, but for now it does not allow the personalization of approaches to the therapy of these diseases. A large number of polymorphisms have been described, the presence of which affects the expression of certain genes associated with the functioning of the immune system, leading to changes in the activity of different parts of immunity. They may occur in various combinations, which would influence the overall structure and functionality of the compromised immune system (62). Thus, it is of interest whether there are common patterns of immunophenotype based on a particular genetic landscape and, if so, whether it is possible to identify for each pattern a causative link that could act as a pathogenetic therapeutic target.

Thus, despite the fact that the value of the peripheral blood immunotype as a biomarker is lower than that of a tissue sample, assessing the general state of the immune system has a number of undeniable advantages. The obvious advantage is the less invasive nature of this approach and possibility to use multiple samples of the peripheral blood for monitoring studies. Also, there are a number of diseases in which taking a biopsy is unnecessary or impossible (type 1 diabetes, juvenile rheumatoid arthritis, multiple sclerosis), where immunotyping of peripheral blood may be an irreplaceable tool for diagnostics. Another advantage is the ability to view the general potential of the immune system to respond to the disease or to the proposed treatment methods, as opposed to the pinpoint picture a biopsy provides. Attempts to establish a relationship between local and systemic immune responses have so far yielded mixed results (63–65). Nevertheless, the articles presented in this review indicate that an individual belonging to a particular immunotype can serve as a reliable biomarker and tool for assessing the potential of the immune system.
